# On the Odours Which Are Exhaled by Living Animals

**Published:** 1801-10

**Authors:** J. J. Virey


### *On the Odours which are exhaled by living Animals.*
### *By* J. J. VIREY.

SINCE the moſt remote ages it has been obſerved that every ſpecies of animal, as well as all the bodies in nature, exhale an odour which is peculiar to them; καὶ ζεύων καὶ φυτῶν καὶ των αψυχων ὅσα οσμωδη. (1.)

If our organs ſcarcely perceive this general property of all beings, it is becauſe they have loſt in infancy great part of their delicacy from the uſe of ſtrong odours, and even too ſavory food. (2.) There have been many inſtances of perſons, however, who have preſerved all the activity and natural extent of their ſenſe of ſmelling in the midſt of ſociety; as the monk of Prague, (3.) who by this means diſtinguiſhed every body, and perceived even, it is ſaid, if a woman was chaſte or not; and we have to regret that death prevented him from finiſhing a Treatiſe on Odours. The celebrated CARDANUS alſo poſſeſſed this ſenſe in a very ſtrong degree; (4.) but it is much more frequently to be met with among men educated in a ſtate of nature, as the ſavage of whom TULPIUS (5.) ſpeaks; the native Americans, (6.) and the negroes of the Antilles, (7.) who can aſcertain by ſmell if their enemies are near or at a diſtance, and whether they are white men or black. (8). Anatomy has accounted for this aſtoniſhing acuteneſs of ſmelling; for SOEMMERING (9.) and BLUMENBACH (10.) have found that the noſtrils are much dilated, and of a great ſize, amongſt theſe nations. Certain inflammatory and ſpaſmodic affections increaſe very much the ſenſibility of this organ, as hydrophobia, (11.) &c. This is in a like manner the caſe with the other ſenſes, which are rendered much more ſuſceptible of the ſlighteſt impreſſion by any ſtate of irritation, on which account Nature ſeems to employ this organization in all her operations that require an increaſe of ſenſibility and irritability. The

(1.) THEOPHRASTUS de Odoribus lib. Daniele Heinſio editore.

(2.) VERDUC Uſage des Parties, tom. II. p. 151. *Taſte influences the power of ſmelling, and vice verſa.*

(3.) Journal des Savans, 1684, in 4to. Paris.

(4.) De Varietate, p. 16. *He was always ſmelling ſomething.*

(5.) Lib. IV. Obſerv. c. 10.

(6.) DIERVILLE, Hiſt. Acad. 1708, p. 120.

(7.) LE CAT Phyſiolog. Treatiſe on the Senſes, p. 256.

(8.) Journal des Savans, 1667, p. 60.

(9.) Anatomical Treatiſe on the Body of Negroes, (in German) p. 22.

(10.) Decas Prima Craniorum Diverſar. gent. tab. IX.

(11.) BORELLUS, Cent. III. Obſerv. Med. 68.

power of habit upon the fenfe of fmelling, difplays itfelf in dogs trained to the chace in fo ftriking a manner, that they diftinguifh the genus and fpecies of animals which they purfue.

The two fenfes of fmell and tafte, fo nearly allied, are the moft fubject of all others to irregularities and idiofyncrafies, which depend, for the moft part, on the conformation of thofe parts. MARTIN SCHURIGIUS has collected many examples of this in his *Chylologia.* It is certain that the fenfe of fmelling, fo juftly denominated by J. J. ROUSSEAU the fenfe of imagination, is one of thofe which has the greateft influence upon the morals and upon the difpofition of mind. (1.) I have, in another work, treated of its great effect on the paffion of love; (2.) and it is eafy to perceive how much it excites and governs it.

As it is not my object to examine this organ of perception, I fhall content myfelf with taking a curfory view of the power of the fmelling faculty in the different claffes of animals. It is obferved, in the firft place, that among thofe animals which are provided with the olfactory membrane, fuch as the *Mammalia*, *birds*, *reptiles*, *and fifhes*, this fenfe feems to be always proportioned to the extent of that membrane. It appears that this property undergoes great modifications in the animals without *vertebræ*, which conftitute the fecond divifion of the animal kingdom; amongft which the olfactory membrane does not exift, though they are not entirely deftitute of the power of fmelling. I have elfewhere remarked, (3.) that, according to my opinion, no animal ought to be wholly deprived of this faculty, fince it would be impoffible for it to feek its nourifhment, and to difcover the prey neceffary to its exiftence; a faculty which is the property of the whole animal kingdom, and without which it would be expofed to deftruction, and the danger of poifon. It has always appeared to me, that among the lower claffes of animals this fenfe is united to that of tafte, as in the greater part of the *Mollusca*, fuch as fnails, mufcles, or even to the fenfes of fight and touch, as in the *Actinæ*, *Medusæ*, the *Polypes* of fweet water, (*Hydræ*, *L.*)

Infects have a very acute fenfe of fmell, which operates at great diftances. It is thus that the *Musca vomitoria*, the *Nicrophores*, *Dermestes*, *Sylphæ*, *Staphylinus*, &c. follow the fmell of putrid

(1.) BENJAMIN RUSH, Medical Enquiries and Obfervations, Vol. ii. p. 34, feq.

(2.) VIREY fur les Philtres, in Magazin Encyclopedique Theomed. Ann. vii.

(3.) VIREY Memoire fur les Vers. Journal de Phyfique, Frimaire, an vii.

putrid animal matters, upon which they feed, and even upon flowers which exhale a ſimilar odour, ſuch as the *Stapelia variegata* and *hirsuta*, the piſtil of the *Dracontium polyphyllum*, *L.* &c.

In general, the ſenſe of ſmelling is inſeparable from that of taſte, of which it ſeems to be merely a modification; nature, therefore, combines them as much as poſſible, becauſe ſhe commonly collects and unites thoſe organs which operate towards the ſame end.

Although cetaceous animals ſeem to be deſtitute of this ſenſe, the *Mammalia pachydeoma* have it in high perfection; vultures too, which feed upon putrid fleſh, ſeem to poſſeſs it in a high degree, as do the owls, the *Buceros*, *L.*; the latter of which have even a double noſe, or a horny naſal tube upon the beak, that increaſes the power of this organ. SCARPA (1) obſerves, that this ſenſe is very conſiderable in the aquatic birds, (*Grallæ*, *L.*) which have long beaks, as the wood-cock, &c. MONRO (2) too aſſures us, that this ſenſe in fiſh is alſo very powerful, conſidering the fluid in which they are immerſed, ſince they can ſmell at a great diſtance the bait which the fiſherman holds out to them. (3.) Though it has been pretended, that the crocodiles power of ſmelling was very delicate, it nevertheleſs appears to be inconſiderable in this animal, as in all other reptiles.

Animal as well as vegetable odours, are naturally arranged under different genera, which, however, it would be difficult to claſs with much preciſion, ſince no objects are leſs capable of diſtinction; we ſhall, nevertheleſs, refer them to five different heads. 1. The muſky or analogous odours. 2. The odores viroſi, like that of caſtoreum. 3. The nauſeous odours, like that of putrid meat. 4. The rancid odours; and 5. The putrid alkaline odours. We ſhall not, however, treat of thoſe which have their origin in the decompoſition of animals, or thoſe parts of them which diſeaſe or death has affected, both becauſe they nearly reſemble each other, and are well known, and becauſe we only intend to examine living bodies.

The general character of animal exhalations is to be fetid, and often ammoniacal. Few acid ſmells are to be met with, except in the claſs of inſects, and particularly of the *hymenoptera*, caterpillars, &c. In obſerving theſe odorous ſubſtances more

---

(1.) Anatom. Obſervat. lib. 2. De Olfactu Diſquiſit. Anat. Paviæ, 1789, fol. c. fig.

(2.) Structure and Phyſiology of Fiſhes. Edinburgh, 1785, fol. c. fig.

(3.) What Dr. Monro attributes to the faculty of ſmelling, may be equally referred to the organ of ſight, after Cit. *Rouſſille Chamſeron.*

more attentively, and even tasting them, it will be perceived, that they are really musky, as one may be easily convinced by that of the ants. (1.) The *carabus crepitans*, and *sclopeta* of Fabricius, emit with noise a similar acid vapour, which must be referred to the *bombic* and *formic acids*. The acid liquor of insects is very corrosive, and they frequently employ it for the destruction of those bodies which Nature has destined to decomposition. One of the perfumes, the most common among the different classes of animals, is the musk odour: Instances of it have even been known in the human species. Plutarch affirms, that Alexander exhaled that smell. Wedelius, and the learned Thomas Bartholin, (2.) have observed the same in several persons: The great Haller (3.) says, that his sweat, and that of many other men, exhibited this phenomenon, and particularly in the morning. Among the apes, the *Simia jacchus*, *L.* and others of that family, produce this perfume; (4.) but it abounds much more among others of the mammalia, as in the opossum, *(Didelphis, L.)* (5.) the *Sorex moschatus*, *L.* (6.) which inhabits the banks of the Wolga, and of the Tanais; of the *Sorex araneus*, *L. &c.* It is on this account that cats only kill, without eating, this animal. (7.)

The irritated hedge-hog, (8.) the *Mus zibethicus* of Gmelin, (8.) the *Mus pilorides*, *L.* (9.) the water rat in the rutting season, (10.) the muscardin, so called from its odour, (11.) and even some hares, according to Conrad, Gesner, and Aldrovandi, exhale this odour chiefly in the rutting season, (12.) or when they are much irritated, a phenomenon that is likewise to be observed in several other animals. The civet is a perfume much resembling

(1.) Fourcroy Philosophe Chimiq. ed. ii. p. 88.

(2.) De Hepate Caustorum, sect. iii. et Desp. Caus. cent. iv. Epist. Med. 87.

(3.) Elementa Physiologia, tom. v. lib. xiv. sect. 2, § 5. p. 163.

(4.) Clusius Exotic. lib. auct. d. Brasilian. Erasm. Franc. p. 386. Hortus Indic. Scaliger de Subtilitate, exerc. 211. § 2.

(5.) Tyson Anat. p. 16. Philosoph. Transact. no. 239, p. 105. Cowper ibid. no. 290.

(6.) Buffon, tom. x. S. G. Gmelin iter liber. p. 28. Guldenstædt's Memoirs of the Berlin Society of Friends of Natural History, (in German,) vol. iii. p. 107, t. 2.

(7.) Ray Quadrup. Synops. p. 239. Pennant's Synopsis of Quadrupeds, p. 307.

(8.) The Calmouks use this animal against the mice instead of a cat.

(9.) Vol. i. p. 125. Schreber Saeugthiere (Mammalia,) tom. iv. p. 638. Sarragin Memoire Acad. Scienc. 1725, p. 323.

(10.) Buffon ibid. p. 2. Rochefort île Antilles, p. 140.

(11.) Recchi Anim. Mexican. p. 587.

(12.) Pallas Glir. Dipos. Method, no. xx. p. 80.

resembling musk, it is produced in a bag under the tail, between the anus and pudenda of the civet cat; *Viverra zibetha*, *L.* (1.) and *Viverra civetta*, *L.* (2.) The genet cat of our southern provinces, *Viverra genetta*, (3.) and that of Malacca, *Viverra malaccensis*, yield also a small quantity of it, the latter of which is much esteemed by the Malays, who use it as a stomachicum and aphrodisiacum. (4.)

GREGORY de BOLIVAR relates, that the sweat of elephants is musky, and that the Indians collect it for the purpose of using it as a perfume. He says the same of the blood of the ox, which, however, is not confirmed by experience. There are, nevertheless, some species of the bull which smell of musk, as the *Bison*, particularly in the rutting season; (5.) and the flesh of the musk ox, *Bos moschatus*, *L.* (6.) The Neapolitan perfumers make use of the dried vulva of the female buffalo in their perfumes. (7.) It has been thought that the same smell was perceptible when the oily flesh of cetaceous animals was roasted. It appears likewise, that the lachrymal glands of the stag and many antelopes exhale a similar odour. (8.)

The animal which furnishes most of this kind of fat perfumed liquor is the musk ox, *Bos msochatus*, *L.* which has been described by a great many authors, the most of whom are noted below. (9.) Travellers have pretended that they procured musk not only from the inguinal bag of that animal, but also

---

(1.) BUFFON, vol. ix. p. 299, t. 31. Mem. Acad. Sc. 1731, p. 443. *Felis Zibethi* Gesner. Quadrup. p 837. Cuvier Tableau des Animaux, p. 123. One obtains more of this substance by irritating it.

(2.) ERXLEBAN Mammifer. BRISSON Regn. Animal. p. 186. HYÆNA et BELON Observat. 208. OLEAVIUS's Voyage, p. 7. Petr. Castellus de Hyæna Odorata, Fr. 1698, in 8vo.

(3.) BELON Observ. p. 73. RIDINGER Illuminate Thiere, i. e. coloured engravings of animals, fig. 28.

(4.) Sencrate Voyage, London, tom. ii, p. 144, t. 91.

(5.) PALLAS Nov. Act. Petropolit. 1777, part iii.

(6.) PENNANT Quadr. p. 27, no. 9. CHARLEVOIX Hist. de la Nouv. France, tom. iii. p. 131. D'OBB's History of Hudf. p. 18.

(7.) Th. Barthol. centur. i. Epist. Med. xlix.

(8.) J. G. GMELIN, it. Sibiric, tom. i. p. 212. PALLAS Spicileg. Zoologiæ i. p. 12. et Miscellan. Zoologica.

(9.) TAVERNIER Voyag. tom. iv. p. 76. THEVENOT. BARBOSA. PAOLO unc. rel. des Indes, p. 216. KIRCHER China Illustr. (translated by Alquic, Amster. 1610, folio.) SCHROEK Historia Moschi Vindobon, 1682, 4to. CHARDIN Voyag. tom. iii. p. 17. CORN. DE BRUYN Voyag. p. 121, tom. ibid. BUFFON, tom. xii. p. 361. NEHEM. GREW Mus. Societ. Londin. 1681, p. 22. sq. PALLAS Spicilegium, Zoolog. Fasc. xiii. tom. iv. GMELIN Nov. Comment. Petropol. tom. iv. p. 393. DAUBENTON Memoires Acad. Science, 1772, p. 215, c. fig.

alſo from the blood, which is made to extravaſate in great ecthymoſes, by means of blows that are given to it. To the tumors thus cauſed, a ligature is applied, by which the blood is dried. TAVERNIER thinks that the whole animal, or, perhaps only its hinder parts, when dried and pulveriſed, are ſold for muſk, which however is improbable. I have found that this perfume triturated with liver of ſulphur became very powerful, in ſuch a degree, that it would not have been ſurpriſing to ſee women, particularly hyſterical and nervous ones, fall into a ſwoon by the ſmell of it, ſince even its ordinary ſmell (1.) as well as that of civet, (2.) is ſometimes ſufficient for that purpoſe. The ſmell of muſk has been known to cauſe a hæmorrhage in a man. (3.) The perfume of the muſk ox is ſtronger the warmer the country which it inhabits; hence the beſt muſk is brought from Tonquin, and the worſt from Syberia, or other cold countries, the perfume of which is ſo diminiſhed, as to approach that of caſtoreum. (4.) Muſk has been counterfeited by a mixture of the bitume of Judea, gummi ſagapenum, galbanum, and opopanax; (5.) a ſimilar odour is likewiſe to be met with in ſome malvaceous plants. It has been aſſerted, that when muſk has loſt its ſmell by long expoſure to the air, it may be reſtored by again expoſing it to the vapour of privies; (6.) and it appears, that human excrements, digeſted and fermented in the balneum mariæ, obtain a muſky ſmell. (7.) FREDERIC HOFFMANN thinks, that this property muſt be aſcribed to the bile which is mixed with it. Dung laid as manure upon the fields has frequently the ſame ſmell. (8.) Stables as well as ſheep cots, and the ſweat of ſheep exhale likewiſe a ſimilar odour. RUELLE formerly remarked, that the excrements of rats were muſky, and uſed to adulterate that ſubſtance. PLINY attributes a muſky quality to the excrements of the crocodile, (9.) which, however, OLAUS BORRICHIUS

---

(1.) LINNE Mater. Medic. Anim. tom. iii. Amoenitat. Academicæ, p. 200, edit. Schreber.

(2.) BOERHAAVE Prælection. Inſt. § 112.

(3.) TRALLES de Limitandis laudib. et Abus. Moſchi, § 3 & 4.

(4.) L. C.

(5.) Hiſt. Acad. Science, 1706, p. 6.

(6.) PAUL AMMANN Manuduct. ad Mater. Medic. § 3, c. 4, p. 191. DANIEL BECKKER Medic. Microcoſm. l. i. c. x. p. 63. ROBERT BOYLE de Utilitat. Experiment. Philoſoph. exercit. x. ſect. ii. § 7, p. 579.

(7.) I. S. ELSHOLZ Deſtillator. Curios. p. 113. AND. LIBAVIUS Griſt. Chemic. 84, lib. ii. p. 50. FRED. HOFFMANN Clavis Schroederi, lib. ii. c. lxxv. p. 106.

(8.) BOYLE Orig. Form. p. 144.

(9.) Hiſtor. Natur. lib. xxviii. c. 8.

Borrichius found not to be always the cafe. (1.) Several ferpents, and particularly the *Natrix Aesculapii* of Laurenti are faid to fpread a fimilar odour. (2.) The flefh and eggs of the crocodile, (3.) as well as its axillary glands, (4.) and feveral fpecies of Oriental and American vipers, (5.) as the rattle fnake, *Crotalus horridus*, *L.* (6.) exhale a very ftrong odour of mufk, which, according to Tyson and Redi, feems to arife from their organs of generation; and it fpreads at a great diftance, fo as to be eafily perceived by the acute fenfe of fmelling of the negroes. Few birds feem to poffefs an odour fimilar to mufk, as the *Anas moschata*, *L.* (7.) and the pectoral skin of the pelican from the Philippines, (8.) which the inhabitants ufe as amulets againft the afthma. An analogous odour is fometimes found in the brown owl, which, however, is too ftrong to be agreeable. (9.)

With regard to the *Mollusca*, it appears that the black juice or ink of the *Sepia* is endued with that odour, as may be perceived in the Indian ink, of which it is the bafis. It is likewife not improbable, that the ambregris found in the ftomachs of the cachelots, *Physeter macrocephalus*, *L.* (10.) originates from the Sepia, the beaks of which are faid to be found in that fubftance. (11.) The operculum of a fnail, *Helix*, *L.* ferved formerly, when previoufly burned, for a perfume under the name of *blatta byzantina.* The fifh *Ougri*, which inhabits the rivers of Brazils, fpreads a mufk odour.

The odour of rofes is to be remarked in feveral fpecies of ichneumon, (12.) but I always found it very difagreeable and acrid. The odour of infects, fuch as the *Apis fragrans*, L. and Fabr. the *Cerambya moschatus*, *Suaveolens*, *Ichneumon mos-*

---

(1.) Sapientia Hermetic. e. x. p. 277.

(2.) Rhodius, centur. iii. Obf. Med. xcviii. Petr. Castellus de Hyæn. Odor. c. 11. Redi Obfervat. Intorno alle Vepere, &c. Laurenti Difp. Amphibior, p. 76.

(3.) Massei Hift. Ind. lib. ii. p. 47. Recchi Hift. Nat. Mexic. lib. ix. c. 3. Labat Afriq. Occid. tom. v. Adanson Voyage au Senegal, &c.

(4.) Labat Ethiop. tom. i. p. 194.

(5.) Hierom Cardan de Subtilit. lib. ix.

(6.) Kirkpatrik de Crotal. ed. ii. p. 15, Anglic.

(7.) Willughby Ornithol. p. 381 & 383.

(8.) Brisson Hift. des Oifeaux, tom. vi. p. 627. no. 3, fol. 46.

(9.) Salerne et Arnault de Nobleville Hift. Nat. Anim. tom. iii. p. 600.

(10.) Schwediaur Philof. Tranfact. 1783, p. 1, no. 15.

(11.) Metzger Ambrologia, et I. F. Klobius Hiftoria Ambaris Wittenberg, 1676, &c. which fhew that Schwediaur's opinion is very old.

(12.) De Geer Mem. pour fervir a l'Hiftoire des Infectes, tom. i. p. 559.

*chator*, *Tipula moschifera*, and many others, may be referred to that of muſk.

An animal exhalation, which ſeems to be fetid at firſt, ſhows frequently by analyſing more accurately by ſmell, ſome analogy with muſk; as, for inſtance, that of an irritated weaſel. The *odor hircinus* and the ſmell of the caſtoreum, are not ſo very different from the former as one might imagine.

It is known that this ſubſtance is found in the anal glands of the *Castor fiber*, *L.* (1.) and the ichorous matter which iſſues from a natural opening on the back of the *Sus tajassu*, *L.* (2.) exhales a very fetid odour of caſtoreum, which is more or leſs obſervable in other ſpecies of ſwine, ſuch as the *Sus Æthiopicus*, which exhibits an odour ſimilar to that of *Lamium purpureum*, *L.*

Many ſpecies of *Geranium* from the Cape of Good Hope, yield an odor hircinus analogous to that of caſtoreum.

[ To be continued. ]

(1.) Rondelet Aquat. p. 236. Gesner Ibid. 185. Buffon, tom. viii. &c.

(2.) Tyson Philoſ. Tranſact. no. 153. Deſmarchais Voyage, tom. iii. p. 296. Bancroft Guian, p. 125.

